# myCOtrak: an app which records smoking, nicotine use and exhaled carbon monoxide concentrations concurrently for use in smoking research

**DOI:** 10.1186/s13104-025-07195-2

**Published:** 2025-04-10

**Authors:** Yue Huang, Joanne Emery, Lisa McDaid, Felix Naughton, Miranda Clark, Anne Dickinson, Sue Cooper, Tim Coleman

**Affiliations:** 1 Centre for Academic Primary Care, Lifespan and Population Health, School of Medicine, University of Nottingham, Nottingham, NG7 2RD UK; 2 School of Health Sciences, University of East Anglia, Norwich, NR4 7UL UK

**Keywords:** Smoking, Nicotine replacement therapy (NRT), CO reading, *NicUse*, *myCOtrak*, *iCOquit*

## Abstract

**Objective:**

Smoking during pregnancy poses significant health risks, necessitating accurate continuous monitoring of pregnant women’s smoking behaviours. Existing methods relying on self-reporting lack objectivity, while biochemical measures like exhaled carbon monoxide (CO) provide validation but suffer from low participant engagement. We developed *myCOtrak* to address these limitations by integrating real-time CO monitoring with self-reported smoking, nicotine replacement therapy (NRT), and e-cigarette use.

**Results:**

*myCOtrak* combines automated CO data from the Bedfont iCO monitor with daily surveys. It demonstrated high feasibility and usability in initial testing with 23 participants, with 75% continuing data submission for ≥ 14 days. Key features include seamless CO integration, cloud-based storage, and longitudinal tracking, offering a validated, scalable tool for smoking cessation research.

**Supplementary Information:**

The online version contains supplementary material available at 10.1186/s13104-025-07195-2.

## Introduction

Smoking during pregnancy remains a major public health concern, linked to complications including preterm birth, low birth weight, and developmental issues, as well as long-term consequences for the child’s health [1, 2]. In the UK, smoking rates during pregnancy have declined, with 7.4% of women in England smoking at the time of delivery in 2023-24 [3]. However, the rate remains high in certain regions and among vulnerable populations, such as younger women and those from lower socioeconomic backgrounds. For instance, some deprived areas in the North West, like Blackpool, have consistently recorded smoking-at-delivery rates double the national average [4].

Many studies evaluate interventions to help pregnant women stop smoking. While some incorporate biochemical verification methods to objectively measure smoking behaviour [5, 6], many still rely primarily on self-reported data, such as surveys or interviews. These methods can be biased by recall and social desirability, which can be particularly problematic during pregnancy due to the stigma associated with smoking. This highlights the need for continuous monitoring of smoking behaviour over time to more accurately evaluate intervention effectiveness [7, 8]. Remote methods for validating self-reported data have become more common but face issues like poor sample return rates [9].

Therefore, we describe how we adapted *NicUse* — a previously developed bespoke data collection app [10] and created the *myCOtrak* app. *myCOtrak* works with the Bedfont iCO monitor [11] to collect and transmit self-reported smoking behaviour data and exhaled CO concentrations to a research database. This method overcomes low return rates associated with validation samples (e.g. saliva) sent by post, and, as it is an impersonal measurement method, it minimises any reluctance to return validation samples related to social desirability bias caused by stigma related to smoking in pregnancy. Furthermore, the app generates time-series data that enable detailed and temporaral behavioural observations to facilitate monitoring and evaluation.

## Main text

### *NicUse*

*NicUse* is a mobile app that collects smoking habits, NRT use, and e-cigarette consumption. The design and pilot testing of *NicUse* is described elsewhere [10]. Evaluated among 35 pregnant women over 28 days, the app demonstrated high usability and acceptability, with 96% finding it easy to use. Data validity was supported by a significant correlation between self-reported smoking behaviours and exhaled CO readings collected independently of the app. This suggests that, while *NicUse* successfully captures self-reported data, incorporating objective measures like CO monitoring could further improve its accuracy in capturing smoking behaviour.

### Bedfont iCO monitor and *iCOquit mums to be*

The Bedfont iCO monitor [11, 12] is a handheld carbon monoxide (CO) device that provides a reliable measure of CO concentrations in expired breath, offering real-time insights into smoking behaviour and exposure to harmful CO, both critical during pregnancy. It operates via the ‘*iCOquit Mums to Be’* app, allowing pregnant women to track their exposure to CO non-invasively.

While the *iCOquit Mums to Be* app allows users to take CO readings, it does not support concurrent data collection on smoking behaviours, NRT use, or e-cigarette consumption. This limitation led to the development of *myCOtrak*, which integrates CO monitoring with comprehensive self-reporting, enabling a more streamlined and holistic approach to tracking smoking behaviour.

### myCOtrak

As mentioned in 2.1, we aimed to extend the uses of *NicUse* by enabling the app to receive CO concentration readings from the Bedfont iCO monitor, and so providing validation for the self-report data. *myCOtrak* was developed from *NicUse* to provide this functionality and works alongside Bedfont’s *iCOquit Mums to Be* App.

**Fig. 1:**
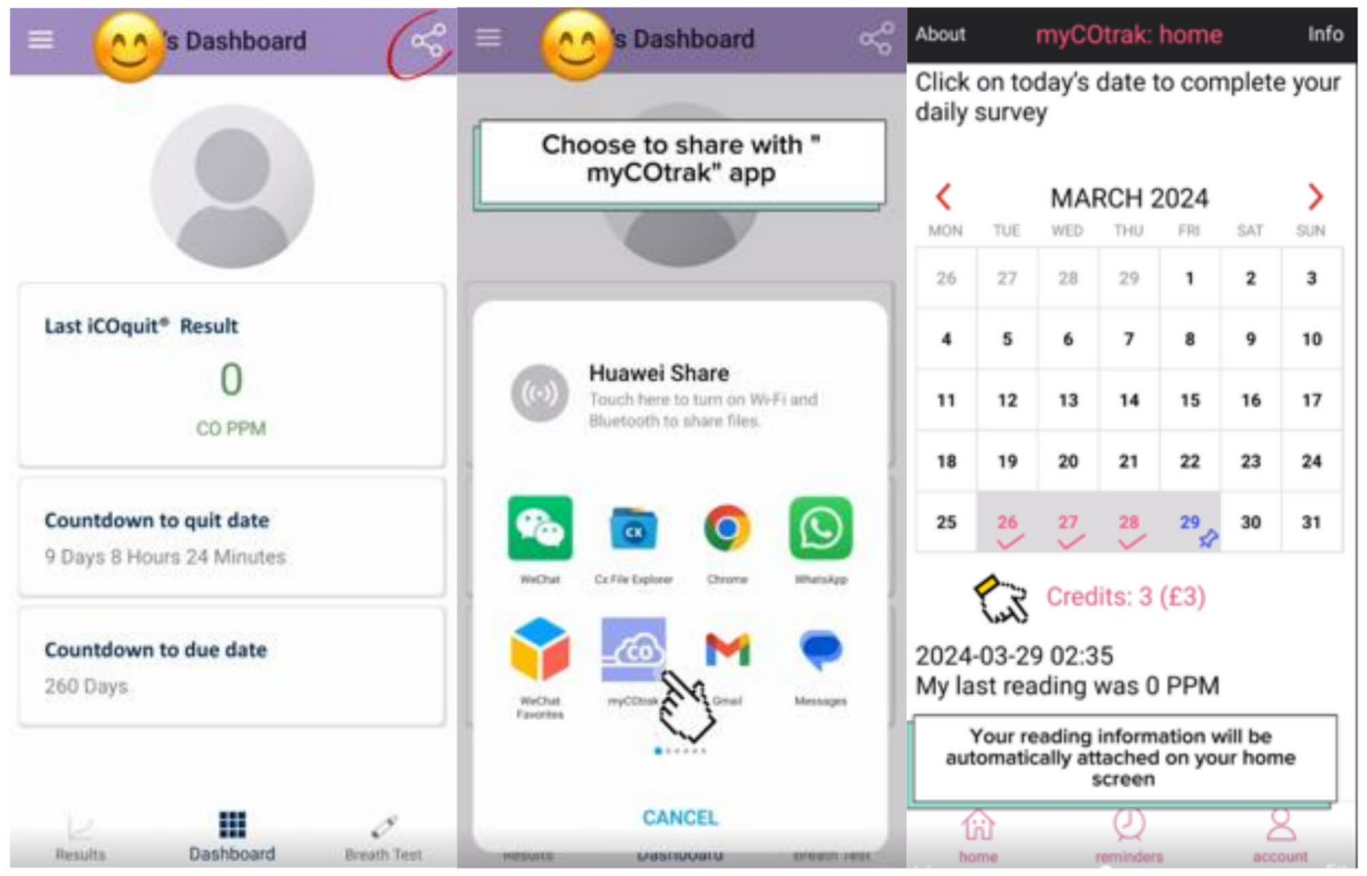
Data sharing between *iCOquit Mums to be* and *myCOtrak*

*myCOtrak* includes financial incentives (currently set at £1 per day), represented as credits in the app, for completing daily surveys, promoting engagement. This addresses a key limitation of existing apps like *iCOquit Mums to Be*, which lack reward-based engagement strategies. Surveys include questions similar to those in *NicUse*, focusing on smoking, NRT, and e-cigarette use in the past 24 h, with some modifications, such as excluding detailed NRT use information in *myCOtrak*. This adjustment was made to simplify the task, ensuring participants can more easily complete the study and remain engaged throughout. Also, this level of detail was not essential for the app’s primary objectives. Clear instructions emphasise that any response earns credits, displayed under a calendar on the summary page (see Fig. 1 below). Participants receive notifications of new credits after each survey submission, which can be redeemed for real rewards like gift cards. Additional features include daily survey reminders and feedback options for the research team, similar to *NicUse* [10, 13].

Figure 1 demonstrates how CO concentrations generated by the *iCOquit Mums to Be* app are shared with *myCOtrak* on Android devices. After logging into the *iCOquit Mums to Be*, the user follows on-screen instructions to provide a CO reading, taps the ‘share’ button in the screen’s top right corner and selects *myCOtrak*. The reading then appears on the *myCOtrak* home screen. The participant can now complete the rest of the survey by selecting the corresponding date on the calendar.

What sets *myCOtrak* apart from *NicUse* is its integration real-time objective measures, rather than relying only on self-reports. By using *myCOtrak* to assess the outcomes of smoking cessation interventions, we aim to find a more effective way to help pregnant women quit smoking.

### How *myCOtrak* works

**Fig. 2:**
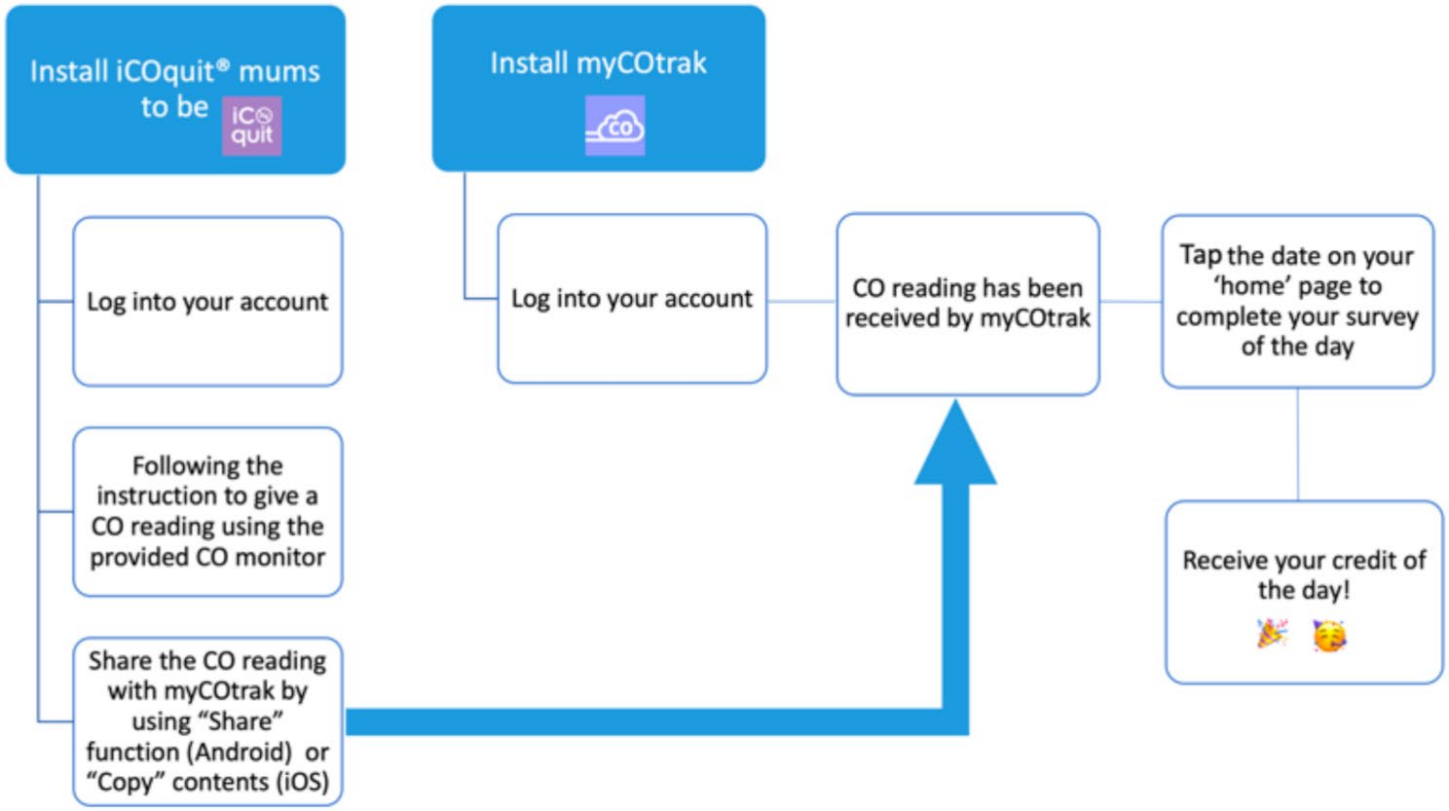
myCOtrak workflow

Figure 2 illustrates how *myCOtrak* is used with the *iCOquit Mums to Be* app and an iCO monitor. Briefly, the user opens both apps, follows standard instructions on the *iCO Mums to Be* app to take a CO reading using the iCO monitor, and then operates the ‘Share’ menu to send the reading to *myCOtrak*.

*myCOotrak* allows for seamless, remote collection of biochemically validated CO readings, taken via the Bedfont iCO monitor, providing a clear and objective measure of smoking status, complementing self-report measures. Since data are collected daily, it holds the potential for exploring longitudinal relationships between smoking patterns and NRT and/or e-cigarette use.

#### *myCOtrak* user interface

**Fig. 3:**
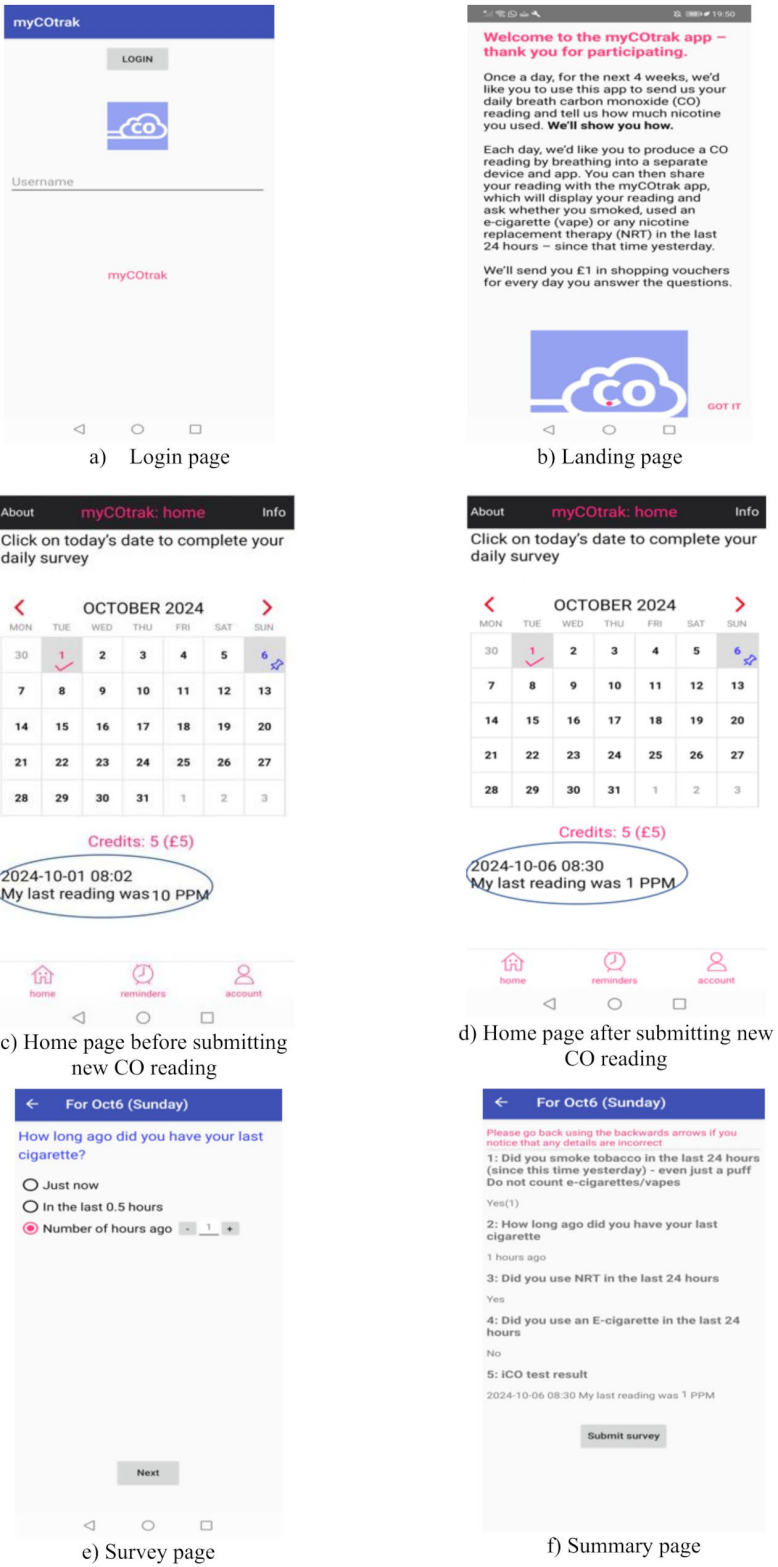
User Interface of *myCOtrack*

Figure 3 illustrates how *myCOtrak* appears on Android devices, with a similar layout on iOS devices. Each user is assigned a unique code for app access. Figure 3a shows the login page; users only need to login once. Figure 3b displays the landing page seen immediately after logging in. Figure 3c shows the home page featuring a calendar. On the calendar, a pink tick below a date signifies that the survey for that day has been completed, while a blue pin indicates an incomplete one. Users tap on a ‘blue pin’ date to start the survey for that day. To ensure that *myCOtrak* measures real time CO concentrations concurrently with self-reported smoking, participants can only complete surveys once on the day that CO readings are collected. Once a reading is shared with *myCOtrak*, the home page updates with the time, date, and CO reading (as highlighted and shown in Fig. 3d). Participants are then prompted to record the number of cigarettes they smoked in the past 24 h for comparison with their CO reading. Figure 3e shows an example survey page, asking participants to report the time since they last smoked and measured their CO level. Users are guided through the entire survey, regardless of their answers. Pop-up instructions are provided to help users complete the survey, while alerts are triggered only in cases of errors, such as lack of signal or submission failures. Before submission of each daily survey and CO concentration, users are prompted to either edit or confirm their answers as correct (Fig. 3f). Full survey questions are in the supplementary document. Once submitted, the data are immediately uploaded to a cloud database [14]. At this point, users can no longer modify the survey responses for that day, and no data are stored on their device.

#### *myCOtrak* researcher interface

The *myCOtrak* researcher interface or web portal is designed for monitoring survey completion, managing participants’ accounts, troubleshooting and collecting user feedback. Survey completion tracking is illustrated in the supplementary document. It allows researchers to manage surveys proactively, enabling them to identify and, if necessary, contact participants who have not completed their surveys. It also allows researchers to flag participants who provide particular responses to specific questionnaire items while the research is ongoing. Surveys can be scheduled to start on specific dates, such as a participant’s smoking quit date.

### *myCOtrak* acceptability testing

Initial *myCOtrak* testing involved four research team members (two male and two female, all non-smokers), using a mixture of Android and iPhones. Testing assessed ease of downloading, in-app text and appearance, and functionality in conjunction with the Bedfont iCO monitor and the app. Team feedback led to minor cosmetic/textual changes to *myCOtrak*. Further testing was carried out with four nonpregnant smokers, who were not research team members, over a two-week period (all female, again using both phone types) to check ease of use and identify any problems. Finally, testing was carried out repeatedly on the same day with one male smoker to check the variation in CO readings.

Currently, a study is underway using *myCOtrak* to collect daily self-reported smoking and other nicotine use data alongside breath CO readings among pregnant/recently pregnant women who smoke. We have recruited 23 participants to date, 19 of whom have successfully downloaded *myCOtrak* and submitted an initial report without a researcher/health professional present, and 15 of whom (> 75%) have continued to provide data for at least 14 days, which is half of project duration, and approximately 58% (11 participants) have inputted data more than 20 days. Results describing the relationship between self-reported smoking and CO readings using *myCOtrak* will be published in a separate paper.

### Conclusions

*myCOtrak* collects accurate, real-time data on smoking status in various contexts, including situations where in-person visits are not feasible. Originally developed for a study on pregnant women, it is adaptable for other groups to monitor changes in smoking behaviour. Its remote, scalable design makes it a valuable tool to maintain continuity in data collection and participant engagement for research and monitoring, especially during situations like the COVID-19 pandemic.

### Limitations

*myCOtrak* offers an objective approach to monitoring daily smoking behaviour, e-cigarette use and other nicotine use, but has some potential limitations:

1. **User Compliance**: Success depends on user engagement. If a project required regular CO readings and self-reports, users would require a high level of adherence, which may vary due to forgetfulness, lack of motivation, or technological difficulties. However, not all studies require repeated measurements. Also, in our experience, a small daily incentive for app completion (e.g. £1 in shopping vouchers per report) appears effective in maintaining participation.
2. **Technical Barriers**: Limited mobile access, poor broadband reception or app usability challenges could reduce *myCOtrak*‘s accessibility among certain populations. Technical issues such as software bugs, connectivity problems, or challenges in syncing with the *iCOquit Mums to Be* device may also reduce its usability. Additionally, the iCO monitor cannot connect directly to *myCOtrak* via the Bedfont API, requiring users to take their CO reading through the *iCOquit Mums to Be* app and then share the reading with *myCOtrak*, before completing the rest of the survey.
3. **Biological Variability**: Environmental factors like second-hand smoke or air pollution can affect CO levels, leading to misinterpretation of smoking status. This could complicate the interpretation of the data, particularly if participants are exposed to high levels of CO exposure from non-smoking sources. We will report the strength of the relationship between self-reported smoking and CO readings obtained via *myCOtrak* in a separate paper as mentioned in Sect. 2.5.
4. **Limited Scope**: Since our study primarily focuses on CO readings, *myCOtrak* may not fully capture all aspects of smoking cessation behaviour, such as the psychological or social challenges involved. Future versions could incorporate additional survey questions on such factors (e.g. mood, environment).
5. **Cost and Resource Limitations**: Providing iCO monitors to all participants and troubleshooting technical issues or replacing faulty devices require significant resources, particularly in large studies.

Despite these limitations, *myCOtrak* represents a significant advancement in remote, objective monitoring of smoking behaviour. Unlike standalone apps that simply transmit CO readings to researchers, *myCOtrak* integrates them with self-reported smoking behaviours in real-time, offering a comprehensive and dynamic view of smoking patterns. We are currently evaluating the feasibility and acceptability of *myCOtrak* for users while also quantifying how well exhaled CO concentrations align with concurrently reported smoking behaviours submitted through the app.

## Electronic supplementary material

Below is the link to the electronic supplementary material.

Supplementary Material 1

## Data Availability

This manuscript focuses on the description of the development and functionality of myCOtrak. Data supporting this study have been collected and are currently under analysis. These data will be made available upon publication of the subsequent results. Requests for access to the dataset can be directed to Dr Yue Huang (yue.huang@nottingham.ac.uk) or Dr Joanne Emery (Joanne.Emery@uea.ac.uk). The dataset includes anonymised self-reported smoking behaviours, NRT/e-cigarette use, and exhaled CO concentrations collected via myCOtrak app.

## Abbreviations

NRT: Nicotine replacement therapy
CO: Carbon monoxide
