# Supplementary material for: myCOtrak: an app which records smoking, nicotine use and exhaled carbon monoxide concentrations concurrently for use in smoking research
Source: BMC Res Notes. 2025 Apr 10;18:158. doi: 10.1186/s13104-025-07195-2 (PMC11987475; doi:10.1186/s13104-025-07195-2)
Supplement: Supplementary file 1 — Supplementary Material 1 [file 13104_2025_7195_MOESM1_ESM.docx]

# Additional file

Add Fig.1 shows how participants’ *myCOtrak* data (self-report and exhaled CO concentrations) are transferred to a Cloud database which researchers can monitor and use.


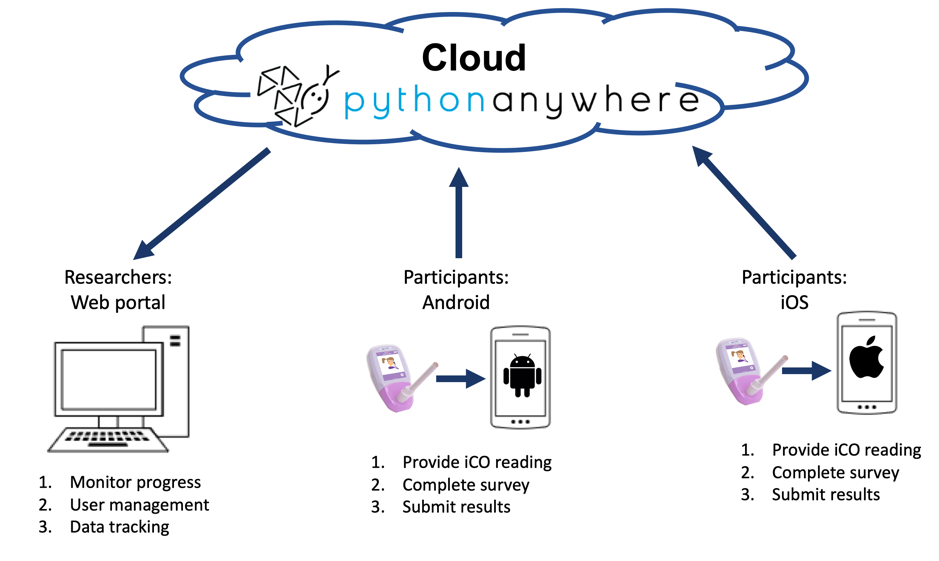
 Add Fig. 1: *myCOtrak* platform

The Cloud used by this app is based on an Amazon web service through Pythonanywhere (<https://www.pythonanywhere.com/?utm_source=chatgpt.com>) as PaaS (Platform as a Service).

Add Fig. 2 illustrates how the survey completion is tracked by the research team. Each cell represents one day and changes from white to purple after the survey of the day is submitted (Add Fig. 2a). By double-clicking on a purple cell, the survey data appears in a pop-up window (Add Fig. 2b).


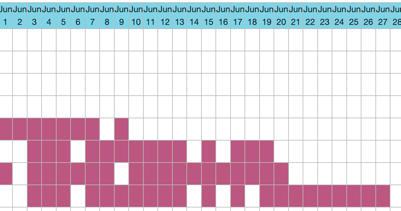


1. Survey progress


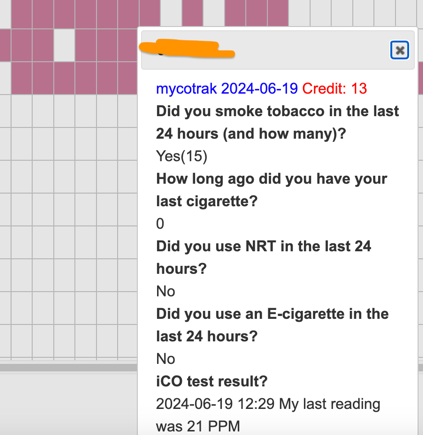


1. Data pop-up when clicking a cell

Add Fig 2. *myCOtrak* web portal for researchers

## The full list of survey questions

1. Did you smoke tobacco in the last 24 hours (since this time yesterday) – even just a puff? Do not count e-cigarettes/vapes.

- Yes (please enter the amount)
- No

1. How long ago did you have your last cigarette?

- Just Now
- In the last 0.5 hours
- Number of hours ago (please enter the number)

1. Did you use NRT in the last 24 hours?

- Yes
- No

1. Did you use an E-cigarette in the last 24 hours?

- Yes
- No
